# Bidirectional Feedback Between Metabolic Reprogramming and Epithelial–Mesenchymal Transition: From Mechanisms to Therapeutic Interventions

**DOI:** 10.3390/molecules31122060

**Published:** 2026-06-12

**Authors:** Yuxin Liu, Mengke Wang, Dan Liu, Hanning Lyu, Deru Zhang, Yang Sun

**Affiliations:** College of Basic Medicine, Heilongjiang University of Chinese Medicine, Harbin 150040, China; 18833575139@163.com (Y.L.); wyyxwmk@163.com (M.W.); ld20190218@163.com (D.L.); lhn6792024@163.com (H.L.); 13942698906@163.com (D.Z.)

**Keywords:** epithelial–mesenchymal transition, metabolic reprogramming, tumor metastasis

## Abstract

Tumor metastasis constitutes a frequent contributor to high mortality rates, with EMT intimately implicated in this disseminative process. Accumulating evidence in recent years indicates that neoplastic cells undergoing EMT frequently exhibit concurrent metabolic reprogramming. Multiple modalities—including glycolysis, mitochondrial oxidative phosphorylation, lipid metabolism, as well as amino acid metabolism—cooperatively supply energy, facilitate membrane remodeling, and sustain redox homeostasis. Specifically, glycolytic flux, oxidative phosphorylation, lipid turnover, and amino acid catabolism/anabolism function in a concerted manner to meet the bioenergetic demands, support biogenesis of cellular membranes, and preserve the intracellular redox equilibrium during phenotypic conversion. Notably, intermediate metabolites can in turn modulate the trajectory of EMT through signal transduction cascades or epigenetic modifications. This review systematically delineates the bidirectional regulatory circuitry interconnecting EMT and metabolic reprogramming; furthermore, it examines the implications of this crosstalk for neoplastic disease progression. Finally, therapeutic strategies targeting the nexus of metabolic reprogramming and EMT are summarized.

## 1. Introduction

### 1.1. Definition of EMT

Epithelial–mesenchymal transition (EMT) represents a critical juncture in the metastatic cascade of tumor cells, during which cells forfeit epithelial attributes while concurrently acquiring mesenchymal characteristics. Cells undergoing EMT exhibit pronounced cytoskeletal reorganization, loss of apical–basal polarity, as well as disruption of intercellular junctions, collectively resulting in diminished adhesive capacity [1]. Molecular hallmarks of this process include downregulation of E-cadherin; in addition, upregulation of N-cadherin and Vimentin occurs. These phenotypic alterations jointly confer enhanced motility upon the cells, culminating in augmented invasive and metastatic potential [2]. As a pivotal regulatory mechanism governing tumor dissemination, EMT plays an indispensable role in the progression of metastasis.

### 1.2. The Concept of Metabolic Reprogramming

The conceptual foundation of metabolic reprogramming originated with the observation of aerobic glycolysis in tumors by Warburg, a phenomenon subsequently demonstrated to be under the governance of oncogenic signals [3]. The classification of metabolic reprogramming as a core hallmark of cancer by Hanahan and Weinberg signified a paradigm shift in the perception of tumor metabolism—from a singular, linear process to a systemic, interconnected network [4]. Driven by oncogenes and compelled by the bioenergetic demands of unchecked proliferation, neoplastic cells remodel the metabolism of glucose, lipids and amino acids [5]. The intermediary metabolites generated during this extensive restructuring can serve as mediators of signal transduction or epigenetic modification, thereby governing malignant phenotypes. Consequently, metabolic reprogramming constitutes a salient feature of tumor metastasis.

## 2. Metabolic Reprogramming and EMT

Cells traversing the EMT process undergo substantial metabolic reconfiguration, a multifaceted adjustment spanning several biochemical domains. These alterations encompass the mobilization of glycogen stores for energy acquisition, modifications in lipid biosynthesis and utilization, as well as shifts in the pathways governing amino acid involvement in cellular activities. Collectively, these adaptive metabolic shifts facilitate the acquisition of migratory and invasive capabilities.

### 2.1. Glucose Metabolism Reprogramming and EMT

#### 2.1.1. Glycolysis and EMT

Glycolysis fulfills a critical function within the tumor microenvironment; its upregulation not only furnishes precursors and bioenergetic substrates for neoplastic proliferation but also engenders a milieu conducive to invasion as well as metastasis [6]. Enhanced glycolytic flux constitutes a cardinal hallmark of metabolic reprogramming in tumor cells, typified by elevated glucose uptake coupled with substantial lactate production, This metabolic pattern directly propels EMT via multiple signaling cascades [7]. Specifically, lactate excreted into the extracellular space acidifies the microenvironment, thereby activating the GPR81 receptor along with hypoxia-inducible factor 1-alpha (HIF-1α). This activation culminates in upregulation of the transforming growth factor-beta (TGF-β)/Smad signaling axis together with core EMT transcription factors, E-cadherin expression declines, intercellular adhesion weakens, and mesenchymal markers such as Vimentin are elevated—collectively augmenting cellular invasiveness and migratory capacity [8,9]. Key glycolytic enzymes—including hexokinase 2 (HK2), phosphofructokinase (PFK), pyruvate kinase M2 (PKM2), as well as lactate dehydrogenase A (LDHA)—exhibit pronounced upregulation, PKM2 can translocate to the nucleus, where it acts as a transcriptional co-activator to directly facilitate the expression of EMT-associated genes [10,11]. This metabolic reconfiguration displays considerable temporal specificity. During TGF-β-induced EMT, glycolytic dependency is not constant; rather, it peaks in both the early and late phases of the process. The early phase relies upon triosephosphate isomerase (TPI), whereas the late phase depends on the activity of energy-investment-stage enzymes such as aldolase, in addition to energy-payoff-stage enzymes like enolase and lactate dehydrogenase. These observations suggest that cells undergoing EMT dynamically adjust glycolytic branch flux to reconcile the competing demands of energy production and biosynthesis [12].

#### 2.1.2. Mitochondrial Oxidative Phosphorylation (OXPHOS) and EMT

OXPHOS exhibits stage-specific functional alterations during the EMT process. Compromised OXPHOS activity can itself serve as a triggering signal for EMT initiation via metabolic-epigenetic crosstalk. Specifically, mitochondrial metabolic reprogramming alters the expression of EMT-related genes through the accumulation of tricarboxylic acid (TCA) cycle intermediates and concomitant chromatin remodeling, ultimately facilitating tumor cell dissemination [13,14]. In ovarian cancer, the transcription factor Ets1 upregulates the expression of the fission protein Drp1, thereby inducing mitochondrial fragmentation and suppressing ATP synthase activity, which in turn promotes EMT [15]. In a sepsis-associated pulmonary fibrosis model, elevated PKM2 mediates both mitochondrial dysfunction and EMT via upregulation of Drp1 coupled with downregulation of mitofusin-2 (MFN2) [16]. In translocation renal cell carcinoma (tRCC), the TFE3 fusion protein binds to genomic regions marked by active chromatin (H3K27ac) to govern the expression of genes implicated in oxidative phosphorylation. This regulation enables tRCC tumor cells to sustain robust OXPHOS activity concurrently with the execution of the EMT program, indicating that heightened OXPHOS metabolism can coexist with the EMT process to collectively drive an aggressive neoplastic phenotype [17]. Conversely, the restoration of mitochondrial function may preserve the epithelial phenotype, the mitochondrially targeted compound mitocurcumin reverses EMT through induction of mitochondrial oxidative stress and subsequent inhibition of the β-catenin/c-Myc axis [18].

#### 2.1.3. The Role of Metabolite-Driven Post-Translational Modifications in EMT

Enhanced glycolysis together with impaired OXPHOS represent two salient perturbations in glucose metabolism reprogramming. This metabolic shift not only alters the predominant mode of cellular energy generation but also frequently results in the aberrant accrual of intermediary metabolites. These metabolites can further promote post-translational modifications (PTMs), thereby modulating the trajectory of EMT at both the epigenetic and signal transduction levels.

##### Lactylation and EMT

Recent investigations have revealed that the driving effect of glucose metabolic reprogramming on EMT is intimately associated with a novel epigenetic modification involving lactate [19]. Neoplastic cells, through heightened aerobic glycolysis (the Warburg effect), engender an abnormally elevated intratumoral lactate concentration [20]. Far from being a mere byproduct of bioenergetic stress, this metabolite functions as a critical signaling precursor that participates in chromatin architectural remodeling. Accumulated lactate is enzymatically converted to lactyl-CoA; under the catalytic action of the acetyltransferase p300, this moiety facilitates histone lactylation at specific lysine residues. For example, lactylation of histone H3 at lysine 18 (H3K18la) has been demonstrated to be precisely regulated by the p300/ASF1A molecular complex [21,22]. Furthermore, the acidic extracellular milieu resulting from high-level lactate efflux can independently potentiate the cascade activation of the TGF-β/Snail signaling axis, establishing a vicious positive feedback loop between metabolic remodeling and signal transduction that further amplifies the invasive and migratory prowess of tumor cells [23]. Research has confirmed that targeted inhibition of PKM2—thereby curtailing lactate production—significantly impedes the EMT process in a colorectal cancer liver metastasis model. This finding substantiates the notion that lactate and its derived lactylation modifications constitute a central molecular nexus linking aberrant glucose metabolism to the malignant behavior of tumor metastasis [24].

##### Glycosylation and EMT

Glycosylation exerts a regulatory influence on the onset of EMT, and a close interrelationship exists between these phenomena during neoplastic progression [25]. The degree of N-glycan branching serves as a pivotal determinant of EMT dynamics. β1,6-linked branching, catalyzed by N-acetylglucosaminyltransferase V (MGAT5), exhibits a positive correlation with tumor malignancy and promotes EMT progression. In contrast, bisecting GlcNAc residues generated by MGAT3 inhibit MGAT5 function, thereby assisting in the maintenance of an epithelial phenotype [26,27]. In hepatocellular carcinoma (HCC), MGAT5 is markedly upregulated in cell lines with high metastatic potential, contributing to N-glycan biosynthesis and facilitating HCC dissemination [28]. During TGF-β1-induced EMT, tumor-derived exosomal miR-663b targets and suppresses MGAT3 expression, consequently advancing the EMT process and metastatic spread [29]. Aberrant glycosylation also plays a significant part in pancreatic cancer progression, MUC16 modified with truncated O-glycans activates focal adhesion kinase (FAK) signaling through an α4β1 integrin complex, thereby enhancing the invasive properties of pancreatic cancer cells [30]. From a metabolic perspective, the heightened glucose uptake characteristic of EMT induction channels a substantial fraction of glucose into the hexosamine biosynthetic pathway. This diversion increases the bioavailability of UDP-GlcNAc, subsequently altering the structural configuration of myriad glycans on the cell surface [31,32]. Concurrently, activated β-catenin during EMT can promote the transcription of the oligosaccharyltransferase complex subunit STT3. Upregulation of STT3, in turn, enhances the N-glycosylation of programmed death-ligand 1 (PD-L1), contributing to the stabilization of this immune checkpoint protein [33].

#### 2.1.4. Targeting Glucose Metabolism as a Therapeutic Strategy

**Figure 1:**
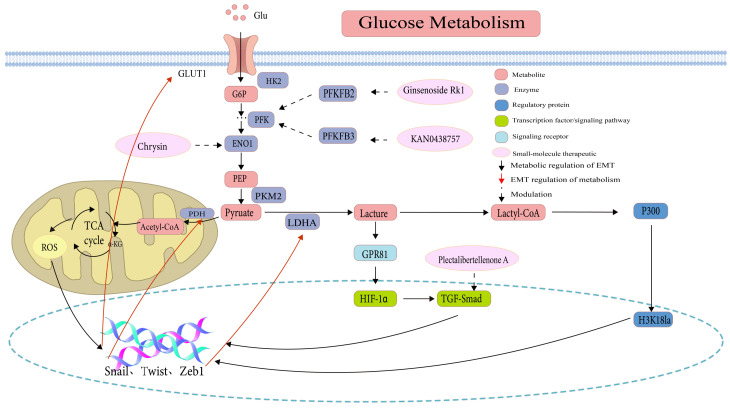
Crosstalk between glucose metabolism reprogramming and EMT.

With regard to glucose metabolism, PFKFB3 functions as a pivotal regulatory enzyme in glycolysis. Its inhibitor, KAN0438757, has been demonstrated to suppress cellular migration while concomitantly reducing N-cadherin expression in glioblastoma—an observation indicative of its potential to target EMT [34]. Ginsenoside Rk1 attenuates aerobic glycolysis via targeted inhibition of PFKFB2. This action curtails STAT3 lactylation along with its subsequent nuclear translocation, culminating in the suppression of EMT and the inactivation of hepatic stellate cells [35]. Chrysin covalently engages α-enolase (ENO1), a critical enzyme within the glycolytic pathway, thereby facilitating its association with β-catenin. This protein–protein interaction promotes the degradation of β-catenin mRNA through an m6A modification-dependent mechanism. Consequently, E-cadherin (CDH1) expression is upregulated, effecting a reversal of the EMT program [36]. Plectalibertellenone A reverses the EMT phenotype in colorectal cancer cells via inhibition of both the TGF-β/Smad and Wnt signaling axes. Furthermore, this compound concurrently suppresses glycolytic flux as well as oxidative phosphorylation, as evidenced by the downregulation of GLUT1, HK2, PKM2 and LDHA expression [37] (Table 1). The key signaling interactions, metabolic intermediates, and drug-targetable nodes in glucose metabolism are summarized in Figure 1.

### 2.2. Lipid Metabolism Reprogramming and EMT

#### 2.2.1. Fatty Acid Synthesis and EMT

During the EMT process, reprogramming of lipid biosynthesis exerts a significant driving force through the activation of pivotal metabolic enzymes, coupled with the regulatory influence of lipid products on cellular architecture and signaling cascades [38]. In cells undergoing EMT, the activities of fatty acid synthase (FASN), acetyl-CoA carboxylase (ACC), and ATP-citrate lyase (ACLY) become aberrantly elevated. These enzymes collectively convert glucose-derived acetyl-CoA into saturated fatty acids such as palmitate, which subsequently serve as precursors for the synthesis of phospholipids, sphingolipids, and cholesterol [39,40]. De novo incorporation of phospholipids into the plasma membrane enhances membrane fluidity and extensibility, thereby facilitating the formation of pseudopodia and filopodia, a transition that drives the morphological shift from an epithelial to a mesenchymal phenotype [41]. Sphingosine-1-phosphate (S1P), a metabolite generated during sphingolipid catabolism, can activate Rac1 as well as Cdc42, orchestrating cytoskeletal rearrangement and directional migration [42]. Excessive synthesis of cholesterol in conjunction with sphingolipids promotes the assembly of lipid rafts. These membrane microdomains provide a platform for the clustering of epidermal growth factor receptor (EGFR) and TGF-β receptors, thereby amplifying PI3K/Akt and Smad signaling axes, which in turn stabilize core EMT transcription factors [43]. TGF-β signaling can indirectly activate sterol regulatory element-binding protein 1 (SREBP1) and its downstream targets, including FASN and ACC, by suppressing negative regulators such as PTEN, thus forging a tight coupling between lipogenesis and the EMT phenotype [44].

#### 2.2.2. Fatty Acid Oxidation and EMT

Fatty acid oxidation (FAO) facilitates the initiation and progression of EMT by supplying bioenergetic substrates, modulating metabolite levels, and influencing critical signaling networks [45]. Carnitine palmitoyltransferase 1A (CPT1A), the rate-limiting enzyme of FAO, catalyzes the conversion of acyl-CoA to acylcarnitine, a prerequisite step for the translocation of long-chain fatty acids across the mitochondrial membrane, upregulation of CPT1A expression consequently enhances mitochondrial β-oxidation, thereby meeting the augmented energy demands of tumor cells during the EMT cascade [46]. Acetyl-CoA generated through FAO can support histone acetylation, elevating the levels of H3K27ac within the promoter region of the EMT transcription factor Zeb1, This epigenetic modification maintains an open chromatin conformation, facilitating Zeb1 expression and perpetuating the mesenchymal cell state [45]. The FAO process also yields reactive oxygen species (ROS), which activate NF-κB and MAPK pathways, further contributing to the stabilization of transcription factors such as Snail [47,48]. Lipid metabolic reprogramming during EMT exhibits dynamic characteristics: anabolic lipid synthesis is primarily governed by SREBP signaling and the PI3K/AKT/mTORC axis, whereas catabolic lipid breakdown is regulated by the AMPK/ACC and SIRT1/PGC1α axes, the accumulation of bioactive signaling lipids constitutes a critical causative factor in propelling the EMT program in tumor cells [49]. Upon uptake into neoplastic cells, fatty acids activate peroxisome proliferator-activated receptors alpha, beta and gamma (PPARα, β/δ, γ), thereby promoting their own uptake as well as mitochondrial β-oxidation in a feed-forward manner. The metabolic restructuring induced by EMT can activate an interactive network involving AMPK, HIF, and mitochondrial respiratory chain complex assembly, thereby coupling mitochondrial respiration with biosynthetic activation to sustain persistent FAO activity [50].

#### 2.2.3. Lipid Signaling Molecules and EMT

Lipid signaling molecules function as second messengers or ligands capable of activating multiple EMT-associated pathways. This action is mediated not through their structural or energetic roles, but rather through signal transduction mechanisms that govern EMT [51]. S1P upon binding to its cognate receptors S1PR1-3, activates the PI3K/Akt pathway, leading to inhibition of glycogen synthase kinase-3 beta (GSK-3β) and subsequent stabilization of Snail with concomitant reduction in E-cadherin turnover. This interaction additionally triggers RhoA/Rock-mediated stress fiber formation and actomyosin contraction, endowing the cell with the contractile, spindle-shaped morphology characteristic of the mesenchymal state [52]. Lysophosphatidic acid (LPA), generated during phospholipid metabolism, engages LPA receptors to activate downstream signals that induce the expression of the transcription factor Zeb1, thereby promoting tumor cell invasion and metastasis. Knockdown of LPA receptors suppresses matrix metalloproteinase-2 (MMP-2) activity, attenuating cellular invasiveness [53,54]. Prostaglandin E2 (PGE2), synthesized via cyclooxygenase-2 (COX-2) catalysis, activates the cAMP/PKA and Wnt/β-catenin pathways through EP receptor engagement. This activation facilitates the nuclear translocation of Snail and Slug while concurrently suppressing the transcription of E-cadherin [55].

#### 2.2.4. Lipid Droplet Dynamics and EMT

Lipid droplets are intracellular organelles responsible for lipid storage and metabolic regulation; their dynamic behavior directly influences the migratory capacity of cells undergoing EMT [56,57]. Perilipin 3 (PLIN3), a lipid droplet coat protein, serves as a critical molecular nexus linking lipid droplet dynamics to the EMT process. Overexpression of PLIN3 in tumor cells promotes lipid droplet biogenesis and stabilizes lipid reserves to sustain energy homeostasis, while simultaneously activating pathways such as Wnt/β-catenin and PI3K/Akt/mTOR to advance the EMT program [58]. Induction of EMT markedly reduces the structural integrity of lipid rafts, a reduction that is essential for the EMT-mediated cellular phenotype. Exogenous supplementation with the omega-3 polyunsaturated fatty acid docosahexaenoic acid (DHA) can restore and reinforce lipid raft stability, thereby inhibiting EMT-driven cell migration [59]. Patatin-like phospholipase domain-containing proteins expressed on the lipid droplet surface, such as PNPLA2 (also known as ATGL), catalyze the hydrolysis of triglycerides and liberate free fatty acids. These free fatty acids participate in membrane lipid metabolism, a process that may indirectly modulate membrane fluidity and cellular locomotion [60].

#### 2.2.5. Lipid Metabolism Remodeling and Ferroptosis Resistance in EMT

Lipid epigenetic remodeling associated with ferroptosis sensitivity represents a salient discovery at the intersection of tumor metabolism and epigenetics. The core mechanism is as follows: to acquire the membrane fluidity and plasticity requisite for migration, mesenchymal-like tumor cells actively alter the fatty acid composition of membrane phospholipids through epigenetic regulation [61]. The EMT master transcription factor Zeb1 differentially governs the expression profile of numerous lipid-metabolizing enzymes. On one hand, Zeb1 upregulates enzymes that facilitate the incorporation of polyunsaturated fatty acids (PUFAs) into membrane phospholipids—including ACSL4, FADS2, and ELOVL5. On the other hand, it suppresses enzymes involved in the generation of monounsaturated fatty acids (MUFAs), such as stearoyl-CoA desaturase (SCD) and FASN. This bidirectional regulatory mechanism elevates the PUFA-to-MUFA ratio within membrane phospholipids, thereby conferring greater conformational freedom upon the plasma membrane, a prerequisite for traversing the physical barriers encountered during metastasis [62]. The accumulation of PUFAs in membrane phospholipids renders cells exquisitely vulnerable to lipid peroxidation, pushing them to the brink of ferroptotic thresholds [63]. To circumvent this endogenous hazard, the EMT program concurrently upregulates ferroptosis defense systems—including glutathione peroxidase 4 (GPX4)—thus maintaining a dynamic equilibrium between survival and invasiveness throughout the metastatic cascade [64].

#### 2.2.6. Targeting Lipid Metabolism as a Therapeutic Strategy

**Figure 2:**
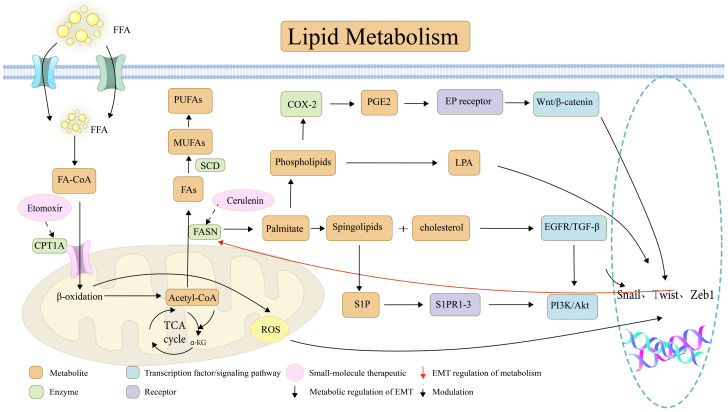
Crosstalk between lipid metabolism reprogramming and EMT.

With respect to lipid metabolism, the FASN inhibitor cerulenin suppresses cellular viability, diminishes intracellular fatty acid levels, and attenuates both the EMT program and stemness-associated phenotypes in glioblastoma. The underlying mechanism entails inhibition of the PI3K/AKT/NF-κB signaling axis coupled with the induction of oxidative stress and endoplasmic reticulum stress [65]. Etomoxir, through suppression of CPT1A activity, impedes the mitochondrial translocation of long-chain fatty acids requisite for β-oxidation. This blockade precipitates intracellular lipid droplet accumulation alongside a decline in ATP levels, thereby activating AMPK while concurrently restraining mTORC1 activity [66] (Table 1). The net consequence is a reduction in the expression of Snail and Twist, which in turn curtails the EMT process in neoplastic cells. The reprogramming of lipid metabolism during EMT, including fatty acid synthesis, oxidation, lipid signaling, and ferroptosis resistance, is illustrated in Figure 2.

### 2.3. Amino Acid Metabolism Reprogramming and EMT

#### 2.3.1. Glutamine Metabolism and EMT

Glutamine metabolism governs the initiation and progression of EMT at multiple levels via its catabolic derivatives—namely, α-ketoglutarate (α-KG), glutamate (Glu) and glutathione (GSH) [67]. The metabolic processing of glutamine is frequently accompanied by the accumulation of ROS, which subsequently induces Snail expression, thereby enhancing the invasive and metastatic capacity of tumor cells [68]. This observation underscores a close association between aberrant glutamine metabolism and the activation of the EMT program. Following cellular uptake, Gln undergoes conversion to Glu and α-KG through the enzymatic activity of glutaminase (GLS). α-KG enters the TCA cycle to replenish carbon intermediates; moreover, it can activate prolyl hydroxylases, thereby facilitating the hydroxylation and subsequent proteasomal degradation of HIF-1α. This process indirectly suppresses the expression of EMT transcription factors Snail and Twist, exerting a negative regulatory effect on EMT [69,70]. In the context of malignancy, however, metabolic aberrations frequently stabilize HIF-1α, paradoxically promoting EMT progression [71]. As an epigenetic cofactor, α-KG modulates the activity of Jumonji domain-containing protein D3 (JMJD3)—an H3K27me3 demethylase—thereby participating in chromatin state regulation, through the removal of H3K27me3 repressive marks, JMJD3 governs the transcription of target genes implicated in the EMT cascade [72]. Furthermore, elevated glutamine availability during EMT can activate the mechanistic target of rapamycin complex 1 (mTORC1) signaling pathway, which contributes to the stabilization of the mesenchymal phenotype [73].

#### 2.3.2. Serine Metabolism and EMT

Serine metabolism facilitates EMT through the synergistic interplay of epigenetic and redox mechanisms, primarily by supplying critical substrates for methylation modifications and antioxidant defense via one-carbon unit metabolism [74]. Through the action of serine hydroxymethyltransferase (SHMT), serine is converted to glycine and 5,10-methylenetetrahydrofolate. Within the mitochondrial compartment, the latter is further metabolized by methylenetetrahydrofolate dehydrogenase 2 (MTHFD2) to generate 10-formyltetrahydrofolate along with NADPH, a crucial cofactor for maintaining redox homeostasis, in the cytosolic compartment, the reductive pathway mediated by MTHFD1 yields the universal methyl donor S-adenosylmethionine (SAM) [75]. HIF-1α directly upregulates the expression of phosphoglycerate dehydrogenase (PHGDH)—the key enzyme of the serine synthesis pathway—as well as the one-carbon metabolic enzyme SHMT2 [76,77]. SHMT2 is typically overexpressed in neoplastic cells and exerts pro-tumorigenic effects; its knockdown markedly attenuates cellular invasion and metastatic potential while concomitantly elevating E-cadherin and reducing N-cadherin levels, indicative of a reversal of the EMT process [78]. Phosphoserine aminotransferase 1 (PSAT1) may be downregulated in colon cancer cells, and its deficiency can activate the PI3K/AKT signaling axis, thereby driving EMT progression and augmenting the migratory and invasive prowess of tumor cells [79]. The one-carbon units supplied by serine metabolism ultimately feed into the methionine cycle, dictating the total flux of methyl donors entering the methionine-SAM axis. The downstream epigenetic ramifications of this flux necessitate examination from the perspective of methionine metabolism [80].

#### 2.3.3. Methionine Metabolism and EMT

Methylation reprogramming mediated by the methionine-SAM axis constitutes a pivotal nexus through which amino acid metabolism regulates EMT. Tumor cells traversing the EMT process exhibit a pronounced dependency on methionine, a reliance that extends beyond its role in protein biosynthesis. As the precursor of the universal epigenetic methyl donor SAM, fluctuations in methionine availability directly influence substrate accessibility for methyltransferase enzymes [81]. Neoplastic cells upregulate the LAT1 (SLC7A5) to scavenge methionine from the tumor microenvironment, thereby preserving intracellular SAM pools [82]. Restriction of methionine leads to a precipitous decline in SAM levels, impairing the catalytic activity of methyltransferases such as enhancer of zeste homolog 2 (EZH2). This impairment results in the erosion of repressive histone marks—most notably H3K27me3—and the concomitant relaxation of chromatin architecture, thereby creating a permissive epigenetic landscape for the expression of EMT transcription factors. A positive feedback regulatory loop exists between LAT1 expression and EZH2 activity: EZH2 alleviates transcriptional repression of LAT1 by inhibiting retinoid X receptor alpha (RXRα), while LAT1-mediated methionine uptake furnishes the requisite methyl donor SAM for EZH2 function [83]. This circuitry amplifies the pro-EMT effects driven by methionine metabolism while simultaneously endowing tumor cells with a survival advantage within nutrient-deprived microenvironments [84].

#### 2.3.4. Targeting Amino Acid Metabolism as a Therapeutic Strategy

**Figure 3:**
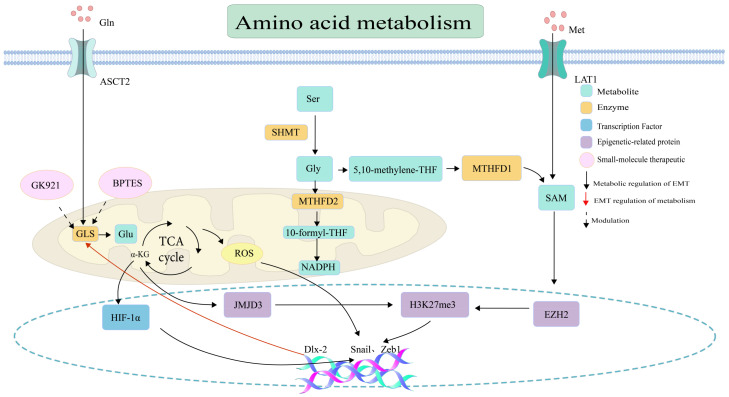
Crosstalk between amino acid metabolism reprogramming and EMT.

With regard to amino acid metabolism, although studies concerning the GLS inhibitor BPTES were reported at an earlier juncture, the principle they elucidated—namely, the selective vulnerability of mesenchymal-phenotype cells to GLS inhibition—retains considerable theoretical significance. Specifically, non-small cell lung cancer cells exhibiting a mesenchymal phenotype, characterized by diminished E-cadherin expression in conjunction with elevated Vimentin levels, display heightened sensitivity to GLS1 suppression. Notably, the induction of EMT can further potentiate cellular susceptibility to GLS inhibitors [85]. GK921, a transglutaminase inhibitor, impedes the EMT cascade, thereby augmenting the chemosensitivity of pancreatic cancer cells to cisplatin and ultimately abrogating tumor cell viability [86] (Table 1). The regulatory network of glutamine, serine, and methionine metabolism in EMT is depicted in Figure 3.

**Table 1:** Small-molecule therapeutics targeting metabolic pathways in EMT.

Small-Molecule Compound	Metabolic Pathway	Tumor Type	Function	References
KAN0438757	Glucose	Glioblastoma	Reduces N-cadherin expression and suppresses cellular migration.	[34]
Ginsenoside Rk1	Glucose	Hepatic Stellate Cells	Inhibits aerobic glycolysis as well as STAT3 lactylation.	[35]
Chrysin	Glucose	Hepatocellular Carcinoma	Promotes β-catenin mRNA degradation and upregulates E-cadherin expression.	[36]
Plectalibertellenone A	Glucose	Colorectal Cancer	Suppresses glycolysis and oxidative phosphorylation; inhibits the TGF-β/Smad and Wnt signaling pathways.	[37]
Cerulenin	Lipid	Glioblastoma	Diminishes intracellular fatty acid levels and inhibits the PI3K/AKT/NF-κB signaling axis.	[65]
Etomoxir	Lipid	Ovarian Cancer	Inhibits CPT1A activity, attenuates fatty acid β-oxidation, and reduces the activity of EMT transcription factors.	[66]
BPTES	Amino Acid	Non-Small Cell Lung Cancer	Inhibits GLS1, thereby disrupting the glutaminolytic metabolic pathway	[85]
GK921	Amino Acid	Pancreatic Cancer	Suppresses the EMT program and enhances cellular chemosensitivity to cisplatin	[86]

### 2.4. Translational Challenges and Strategic Opportunities

The small molecule inhibitors currently under investigation have demonstrated promising preclinical efficacy in targeting metabolic nodes to inhibit EMT and tumor metastasis; however, advancing these agents to clinical application still encounters several translational barriers. Most metabolic enzymes, such as FASN, CPT1A and GLS1, are also widely expressed in normal tissues, which may result in off-target inhibition of these enzymes in normal somatic cells when the drugs are aimed at tumor cells, thereby causing toxicity issues. Due to the metabolic plasticity of tumor cells, when a specific metabolic pathway is obstructed by a drug, these cells can shift their energy sources to alternative metabolic pathways, thereby evading the cytotoxic effects of a single agent. Therefore, the combined targeted inhibition of multiple metabolic pathways may prove more effective than monotherapy [87]. The context-dependent nature of metabolic vulnerabilities also suggests that patient stratification based on tumor type, EMT status, and metabolic biomarkers is critical for clinical success. Furthermore, the combination of metabolic inhibitors with immune checkpoint blockade or traditional chemotherapy may yield synergistic effects, as metabolic reprogramming significantly influences the tumor immune microenvironment through mechanisms such as lactate accumulation and pH homeostasis regulation [88]. It is essential to develop more selective inhibitors of metabolic enzymes and conduct biomarker-driven basket trials to identify patients who are likely to benefit from these therapies.

In summary, the reprogramming of glucose, lipid and amino acid metabolism does not occur in isolation; rather, these three metabolic axes operate in a concerted and synergistic manner to influence the EMT process. Enhanced glycolysis within carbohydrate metabolism serves as the cornerstone, rapidly furnishing ATP and glycolytic intermediates. This metabolic mode directly activates EMT transcription factors such as Snail and Zeb1 while simultaneously modulating E-cadherin stability through lactate-mediated epigenetic modifications and Acetyl-CoA-dependent protein glycosylation, thereby establishing the bioenergetic and signaling foundation for EMT. Lipid metabolism, through the dynamic equilibrium between fatty acid synthesis and oxidation, supplies the phospholipids and signaling molecules essential for membrane remodeling in mesenchymal cells. Concurrently, NADPH and Acetyl-CoA generated via FAO play indispensable roles in preserving redox homeostasis and sustaining histone acetylation, thus supporting cellular morphological extension and migratory capacity. Amino acid metabolism contributes by anaplerotically replenishing the TCA cycle, providing nitrogen sources and nucleotide precursors, in addition to modulating the levels of metabolites such as α-KG and succinate to influence the activity of DNA and histone demethylases. Collectively, this orchestrated interplay—whereby glycolysis provides energy, lipid metabolism constructs membranes, and amino acid metabolism modulates signaling cascades—collaboratively propels the transition from an epithelial to a mesenchymal state. This metabolic synergy furnishes the metabolic plasticity and microenvironmental adaptability essential for tumor invasion, metastasis and therapeutic resistance. Targeting critical nodes within this integrated metabolic axis holds considerable promise for suppressing EMT-driven neoplastic progression.

## 3. Reciprocal Regulation of Cellular Metabolic Reprogramming by EMT

During the execution of the EMT program, neoplastic cells acquire enhanced migratory and invasive capabilities; moreover, EMT exerts a profound influence on the cellular metabolic network. Diverging from earlier unidirectional models that predominantly emphasized metabolic reprogramming as a driver of EMT, recent investigations have revealed that the EMT state can conversely modulate metabolic pathways. Through the regulatory actions of specific transcription factors, EMT alters the expression of relevant metabolic enzymes and modifies mitochondrial function, thereby reshaping the epigenetic landscape and establishing a metabolic configuration conducive to the mesenchymal state.

### 3.1. EMT and Glucose Metabolism

While propelling tumor metastasis, the core transcription factors governing EMT simultaneously remodel the cellular metabolic circuitry. Snail, Zeb1, and Twist act in a concerted manner to potentiate the Warburg effect at multiple levels [89]. Snail, a core transcription factor governing EMT, can bind directly to the promoter region of fructose-1,6-bisphosphatase (FBP1)—the rate-limiting enzyme in gluconeogenesis—thereby repressing its transcriptional activity and leading to downregulation of FBP1 expression. Restoration of FBP1 expression, conversely, suppresses the EMT program [90]. Aldehyde dehydrogenase 1A3 (ALDH1A3) functions as a critical molecular switch governing the transition between EMT status and glucose metabolic mode in breast cancer stem cells. By promoting OXPHOS while significantly diminishing glycolytic activity, ALDH1A3 confers upon epithelial-like breast cancer stem cells a metabolic signature characterized by low glycolysis and high OXPHOS. Knockdown of ALDH1A3 reverses this phenotype, redirecting cellular dependence toward glycolytic metabolism [91].

### 3.2. EMT and Lipid Metabolism

At the level of lipid metabolism, EMT transcription factors inversely drive lipid metabolic reprogramming through direct transcriptional control of lipid-associated genes. The principal hallmarks of this reprogramming are the suppression of de novo fatty acid synthesis alongside the enhancement of FAO [92]. EMT transcription factors downregulate the expression of multiple lipogenic genes, including FASN and ACC, thereby curtailing fatty acid biosynthesis [93]. Mesenchymal cells may compensate for this reduction through heightened uptake of exogenous lipids via transporters such as CD36, displaying a pronounced reliance on FAO to satisfy bioenergetic demands [94]. The EMT transcription factor Zeb2 directly engages and activates ACSL4 expression; ACSL4, in turn, promotes lipid droplet accumulation and potentiates FAO, furnishing the energetic foundation requisite for tumor cell dissemination [95]. This dual mechanism—attenuated anabolism coupled with augmented catabolism—both meets the material demands of heightened membrane fluidity essential for mesenchymal cell motility and provides energy reserves, in the form of accumulated lipid droplets, to buffer against metabolic stress.

### 3.3. EMT and Amino Acid Metabolism

The EMT transcription factor Dlx-2 directly upregulates the expression of GLS, thereby enhancing the conversion of Gln to Glu and providing anaplerotic carbon sources for the TCA cycle [96]. Concomitant with this shift, the utilization of Gln transitions from predominant GSH synthesis for redox homeostasis toward reductive carboxylation pathways that supply acetyl-CoA for lipid biosynthesis [97]. During EMT progression, the loss of the epithelial-specific transcription factor ELF3 leads to diminished epithelial identity, permitting ATF4 binding within mesenchymal genomic regions to activate the transcription of genes encoding aminoacyl-tRNA synthetases and related stress-response elements. This regulatory switch—from ELF3-mediated repression to ATF4-driven activation—directly couples amino acid deprivation signals to the EMT transcriptional program [98]. The resultant amino acid metabolic remodeling, orchestrated by EMT transcription factors, supplies the carbon skeletons and nitrogen sources essential for rapid mesenchymal cell proliferation and migration. Simultaneously, this reprogramming engenders a distinct metabolic vulnerability by diminishing endogenous antioxidant capacity, thereby presenting a potential therapeutic window for the selective targeting of mesenchymal-like tumor cells [99].

### 3.4. Metabolic Rewiring and EMT Plasticity: A Context-Dependent Interplay

The bidirectional regulation between EMT and metabolic reprogramming is not a fixed program; rather, it exhibits significant context dependence across various tumor types, genetic backgrounds, and microenvironments. In breast cancer, EMT is closely associated with enhanced glycolysis and the upregulation of glucose transporters such as GLUT3 and GLUT12 [100]. Conversely, in gastric cancer, tumor tissue-derived mesenchymal stem cells display a pronounced reliance on FAO, with CPT1A-mediated FAO metabolic reprogramming directly supporting the lymphatic metastasis capabilities of gastric cancer cells [101,102]. Notably, cells undergoing EMT do not exist solely in the “epithelial” or “mesenchymal” states; there are also intermediate transitional forms characterized by high plasticity. Bocci et al. demonstrated that the transcription factor NRF2 prevents cells from undergoing a complete EMT by inhibiting classical EMT transcription factors such as Snail. This inhibition stabilizes cells in a hybrid epithelial/mesenchymal phenotype, with NRF2 expression peaking in this phenotype, thereby revealing a direct molecular link between metabolic regulators and EMT plasticity [103]. Recognizing this context dependency is crucial for developing precision therapies that target metabolic vulnerabilities in a tumor type- and EMT state-specific manner.

In summary, EMT actively reprograms the metabolic network through the concerted actions of transcription factors, epigenetic modifications and microenvironmental cues. This transformation shifts the cellular metabolic paradigm from an epithelial configuration—dominated by oxidative phosphorylation—toward a mesenchymal state characterized by heightened glycolysis, dynamic lipid synthesis and oxidation, as well as glutamine dependency. Such reciprocal regulation of metabolism by EMT constitutes a fundamental underpinning of tumor adaptation, metastatic dissemination, stemness maintenance and therapeutic resistance.

## 4. Concluding Remarks and Future Perspectives

Although significant progress has been made in recent years regarding the bidirectional regulation between metabolic reprogramming and EMT, several key knowledge gaps remain in this field. The causal relationship between metabolic alterations and EMT is still unclear; most existing studies have reported correlations between certain metabolic phenotypes and mesenchymal states, but direct genetic evidence demonstrating that manipulation of a single metabolic enzyme is sufficient to drive or reverse EMT in vivo is lacking. Furthermore, the non-canonical functions of metabolic enzymes in EMT have been severely underestimated. The discovery of nuclear translocation of PKM2 acting as a transcriptional coactivator has revealed the “moonlighting” functions of metabolic enzymes. However, whether other key enzymes such as fatty acid synthase and glutaminase possess similar mechanisms and directly participate in the EMT transcriptional regulatory network remains under-researched [104]. Additionally, the metabolic heterogeneity of hybrid or intermediate EMT states remains largely unexplored, as the vast majority of studies have only compared the two endpoints—pure epithelial and fully mesenchymal—while ignoring the widespread existence of partial EMT states, which may possess unique metabolic vulnerabilities [105]. The specificity of metabolic dependencies related to tumor type and genetic background remains unclear, and findings derived from breast cancer model may not be directly applicable to other malignancies [106]. Differences in EMT-associated metabolic reprogramming patterns may be fundamental between various tumor types and among distinct genetic subtypes of the same tumor. The mechanistic details of the metabolic-epigenetic regulatory axis are still poorly understood. While metabolite-driven epigenetic modifications, such as lactylation, acetylation, and methylation, have been confirmed to play a role in EMT regulation, the dynamic patterns of these modifications during EMT, the specific modification sites, and the coordination mechanisms of the upstream and downstream regulatory networks require further in-depth elucidation [107].

Based on the identified knowledge gaps, this paper proposes several future research directions that warrant focused attention. In the context of drug development, the creation of highly selective metabolic enzyme inhibitors is a primary objective for clinical translation. Future efforts should concentrate on developing isoform-selective inhibitors to enhance the therapeutic window. Additionally, novel degradation technologies, such as proteolysis-targeting chimeras, present opportunities for increased selectivity in targeting metabolic enzymes and overcoming compensatory upregulation. Regarding biomarkers, the identification of predictive biomarkers to guide patient stratification is essential. Potential biomarkers include the lactylation levels in tumor tissues, the expression levels of CPT1A, the ratio of polyunsaturated to monounsaturated fatty acids in membrane phospholipids, and the expression profiles of key metabolic enzymes. Concerning technical approaches, the utilization of spatial metabolomics, in vivo stable isotope tracing, and CRISPR-based metabolic gene screening will facilitate a systematic dissection of the causal relationship between metabolism and EMT. Finally, targeting metabolic-epigenetic crosstalk represents a promising and emerging therapeutic avenue. The development of small molecule compounds that target epigenetic modifying enzymes and their associated reader proteins, while simultaneously addressing the coupling between metabolite supply and the activity of these enzymes, may effectively inhibit tumor metastasis by reversing EMT-related epigenetic reprogramming.

## Data Availability

No new data were created or analyzed in this study. Data sharing is not applicable to this article.
